# Report of a Rare Case and Literature Review of Combined Marcus Gunn Jaw Winking Synkinesis and Monocular Elevation Deficiency

**DOI:** 10.7759/cureus.88119

**Published:** 2025-07-16

**Authors:** Omar Moussa Pasha, Rocio Bentivegna, Gabriela Espinoza

**Affiliations:** 1 Department of Ophthalmology, St. Louis University School of Medicine, St. Louis, USA

**Keywords:** amblyopia, double levator palsy, marcus-gunn jaw winking synkinesis, monocular elevation deficiency, strabismus

## Abstract

Marcus Gunn Jaw Winking Synkinesis (MGJWS) is a rare congenital condition characterized by unilateral ptosis that improves with ipsilateral jaw movement. It frequently presents alongside other ocular abnormalities, including strabismus and anisometropia, which significantly increase the risk of amblyopia. Monocular elevation deficiency (MED), a type of strabismus marked by impaired elevation of the eye in all gaze positions, is most commonly associated with MGJWS. We report the case of a 14-year-old male with a history of refractive amblyopia who presented with both MGJWS and MED. This case supports emerging evidence that MGJWS and MED may represent phenotypic variations of the same neurodevelopmental disorder, with the severity of ptosis possibly potentiating the co-occurrence of MED. It also underscores the importance of early and comprehensive ophthalmologic evaluation in patients with MGJWS for the detection of coexisting ocular abnormalities that increase the risk of amblyopia.

## Introduction

First described by Scottish ophthalmologist Robert Marcus Gunn in 1883, Marcus Gunn Jaw Winking Synkinesis (MGJWS) is a rare congenital condition characterized by unilateral ptosis that improves with stimulation of the ipsilateral pterygoid muscles. It occurs in 2% to 13% of patients with congenital ptosis and presents as elevation of the ptotic eyelid during movements such as chewing or mouth opening [1]. The mechanism behind MGJWS results from misrouted signals from the trigeminal nerve, whereby motor impulses intended for the external pterygoid muscle are misrouted to the levator palpebrae superioris muscle, leading to involuntary eyelid elevation during jaw movement [2]. Although MGJWS may present as an isolated finding, it more often presents alongside other ocular abnormalities like strabismus or anisometropia, many of which are associated with an increased risk for the development of amblyopia [3].

The risk of the development of unilateral amblyopia, defined as decreased visual acuity in one eye caused by abnormal binocular interactions during the early stages of visual development, is compounded by the presence of strabismus or other refractive errors. In one study, strabismus was associated with a 2.7 to 18 times greater odds of amblyopia development when present [4,5]. One type of strabismus, monocular elevation deficiency (MED), is characterized by the inability to elevate one eye in abduction, adduction, and primary gaze [6,7]. The pattern of gaze limitations seen in isolated MED is theorized to occur through either thickening of the inferior rectus muscle or through disruptions to the unilateral center for upgaze, with the oculomotor nerve being functionally intact for most patients [6]. Though the underlying pathophysiology is not fully understood, MED frequently co-occurs with MGJWS and represents the most common type of strabismus observed, with studies reporting a prevalence of 25% to 48% among patients with the condition [3,8].

Given the extensive overlap between MGJWS, MED, and other ocular abnormalities that increase the risk of amblyopia development, the presentation of MGJWS in a patient should prompt further investigation into other possible irregularities for amblyopia risk stratification. Thus, to further shed light on the association between MGJWS and accompanying ocular abnormalities, we present the case of a 14-year-old male with a history of refractive amblyopia who presented to the ophthalmology clinic with MGJWS and MED.

## Case presentation

**Figure 1:**
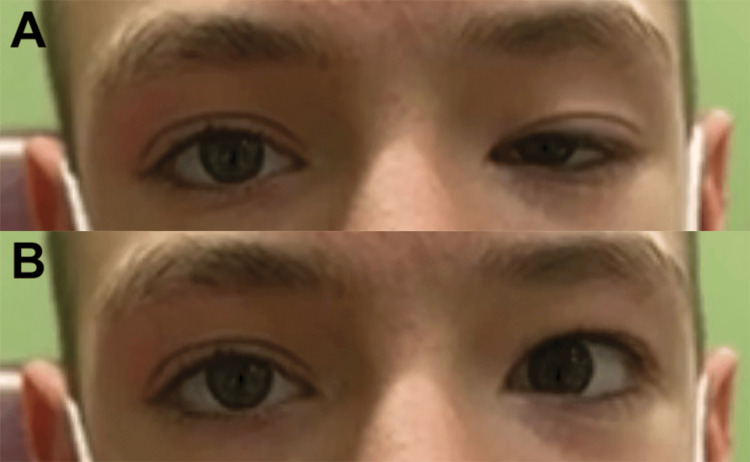
Preoperative evaluation of MGJWS Image A was taken at rest. Image B was taken during active jaw movement. MGJWS: Marcus-Gunn jaw winking synkinesis

**Figure 2:**
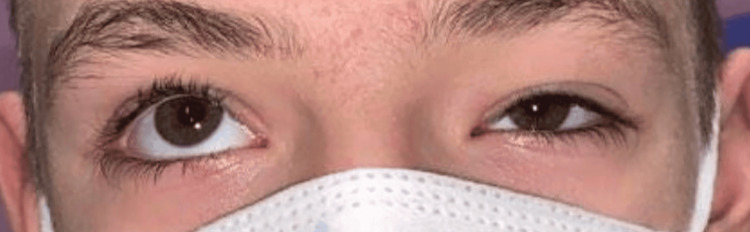
Preoperative evaluation of MED in primary gaze MED: Monocular elevation deficiency

A 14-year-old male with a history of refractive amblyopia was referred to the ophthalmology clinic for evaluation of left upper eyelid ptosis, which has been present since birth. Slit-lamp examination revealed a palpebral fissure height of 8 mm in the right eye and 3 mm in the left, with marginal reflex distance (MRD) measurements of +3 mm in the right eye and -2 mm in the left. Notably, the patient’s ptosis improved significantly when he opened his mouth, as illustrated in Figure 1. Upward gaze in the left eye was also restricted in all gaze positions, as shown in Figure 2. No evidence of esotropia or epiblepharon was observed on examination. Given the patient’s restricted upward gaze and ptosis that improved with jaw movement, these findings were suggestive of MGJWS with accompanying MED.

Due to a good levator function of 12 to 13 mm in the left eye and a negative Hering’s law response, the patient initially underwent levator advancement surgery. At the one-week postoperative visit, the MRD improved to +2 mm. However, due to persistent ptosis, a frontalis sling procedure was subsequently performed. One week following the second surgery, the patient exhibited minimal evidence of MGJWS, which was only apparent with effort. Residual ptosis persisted, with an MRD of 0 mm at rest and +2 mm during brow activation, though the accuracy of the examination was limited by expected postoperative edema. Unfortunately, further assessments could not be performed, as the patient was lost to follow-up.

## Discussion

Although the association between MGJWS and MED is well-documented, the underlying mechanism connecting them remains unclear. Shahabinia et al. suggest that this relationship may be explained within the broader framework of congenital cranial dysinnervation disorders (CCDDs), a spectrum of congenital, non-progressive conditions with a common underlying etiology [9]. According to their hypothesis, a developmental failure in the proper innervation of the levator palpebrae superioris and the superior rectus muscle, both normally innervated by the superior branch of the oculomotor nerve, results in congenital ptosis and MED, respectively. Concurrently, this disruption may also lead to MGJWS by inducing aberrant synaptic connections between the oculomotor and trigeminal nuclei due to their anatomical proximity during early neurodevelopment. This mechanism elegantly explains the simultaneous manifestation of MGJWS and MED, reinforcing the hypothesis that they represent varying manifestations of a single neurodevelopmental disorder. 

Furthermore, a pattern emerging from recent case reports suggests that the severity of ptosis may predict the presence of MED in patients with MGJWS. Shah et al. described an 18-year-old male with bilateral MGJWS, in whom the eye with more pronounced ptosis also exhibited MED [10]. Another report by Shahabinia et al. described a 13-year-old girl with severe congenital ptosis who had both MGJWS and MED, whereas her aunt, who had milder ptosis, exhibited MGJWS without MED [9]. Our case aligns with this trend because the patient’s severely ptotic eye, evident by MRD -2, was affected by both MGJWS and MED. This observed correlation strengthens the hypothesis that MGJWS and MED may not be distinct conditions but rather varying phenotypic expressions of a single neurodevelopmental disorder. The dual manifestation likely reflects the degree of dysinnervation, whereby more severe disruptions of oculomotor nerve development increase the chance of forming aberrant oculomotor-trigeminal synapses, leading to the dual presentation of MGJWS and MED.

This report aims to highlight the frequent association between MGJWS and co-existing conditions such as strabismus, particularly MED, and anisometropia, both of which are strongly linked to the development of amblyopia [3]. In fact, the incidence of amblyopia has been reported in 35% to 59% of patients with MGJWS, underscoring the need for early detection and management [8,11]. Given this strong association, clinicians should maintain a high index of suspicion for these abnormalities when evaluating patients with MGJWS to prevent amblyopia and its long-term consequences. Effective management requires a comprehensive ophthalmologic assessment with careful consideration of the severity of ptosis and the extent of jaw-winking [9]. Patients should also be counseled on the potential need for multiple surgeries to achieve satisfactory cosmetic outcomes, as demonstrated in this case.

In addition, this report explores the current literature on the possible pathophysiological link between MGJWS and MED. Although data remain limited, emerging evidence suggests that both conditions may represent varying phenotypic expressions of the same neurodevelopmental disorder belonging to a group of conditions referred to as CCDDs [9,12]. Further studies are needed to verify these hypotheses with the goal of improving our understanding of these conditions and optimizing treatment strategies to minimize the cosmetic, visual, and surgical burden on affected patients.

## Conclusions

This report reinforces the well-documented association between MGJWS and MED. It also supports the hypothesis that these conditions likely represent different manifestations of the same neurodevelopmental disorder. Early and comprehensive ophthalmologic evaluation is critical in patients with MGJWS to detect and address coexisting conditions, such as strabismus and anisometropia, before the onset of amblyopia. Further research is needed to elucidate the underlying pathophysiological mechanisms linking MGJWS and MED to enhance our understanding of these conditions and develop optimized management strategies that improve patient outcomes.
